# Ocular Syphilis: Our Experience in Selayang Hospital, Malaysia

**DOI:** 10.7759/cureus.26655

**Published:** 2022-07-08

**Authors:** Nur Izzati Mohd Fadzil, Abbas Abd Hamid, Julieana Muhammed, Hanizasurana Hashim

**Affiliations:** 1 Department of Ophthalmology, Selayang Hospital, Selangor, MYS; 2 Department of Ophthalmology and Visual Science, School of Medical Sciences, University of Science Malaysia, Kelantan, MYS

**Keywords:** treponema pallidum, msm, ocular syphilis in asia, syphilitic uveitis, hiv, ocular syphilis

## Abstract

Objectives: This study aims to describe the demographic features, clinical profile, Human Immunodeficiency Virus (HIV) status, and visual outcome after completing treatment in patients diagnosed with uveitic syphilis.

Methods: A retrospective review was conducted of all cases diagnosed with ocular syphilis from January 2014 to December 2019 at the ophthalmology clinic of Selayang Hospital, Selangor, Malaysia. A total of 31 cases were reviewed, and the collected data included demographic features, history of high-risk behavior, ocular symptoms and signs, visual acuity at presentation and after completing treatment, treatment received, complications, and HIV status. Serology tests to confirm the diagnosis were also included, such as the rapid plasma reagin (RPR), venereal disease research laboratory test (VDRL) titer, and treponema pallidum hemagglutination (TPHA) tests, and some cases also included the VDRL cerebrospinal fluid (CSF) test.

Results: A total of 31 patients with ocular syphilis were identified within the study period. Male patients comprised the majority with 27 cases. Nineteen patients were below the age of 50. The majority were ethnic Malay (21 patients). Seventeen patients were identified to have HIV co-infection. Twenty patients reported high-risk behaviors, and among them, six cases were HIV-infected homosexuals. The commonest symptom was blurring of vision (61%), followed by eye redness (16%), floaters (13%), and incidental findings (10%). There were 18 bilateral cases and 13 unilateral cases. The larger share presented as panuveitis (14 cases), followed by intermediate uveitis (nine cases), anterior uveitis (four cases), posterior uveitis (two cases), endophthalmitis (one case), and branch retinal vein occlusion (one case). RPR and TPHA tests were done for all patients. Only 12 patients consented to lumbar puncture for a CSF VDRL test, and one tested positive. All patients received intravenous (IV) administration of 3.0 to 4.0 million units of benzylpenicillin every four hours for 14 days. All cases reported a good outcome with an improvement in visual acuity of at least two Snellen lines after treatment.

Conclusion: Early detection and treatment of ocular syphilis will usually preserve visual acuity and ocular function. This study highlights the need for a high degree of suspicion of HIV co-infection, as the majority of our patients were discovered to be HIV-seropositive. Thus, HIV screening is mandatory in all patients presenting with syphilitic uveitis.

## Introduction

Syphilis is a type of sexually transmitted infection caused by an organism called *treponema pallidum*. The prevalence is higher in men who have sex with men (MSM) [1]. In Asia, there is limited data due to cultural beliefs in Asian countries. The latest data available from WHO reports that the prevalence of syphilis in Southeast Asia was about 5.9 cases per 100,000 in 2014 [2].

Ocular syphilis can affect most structures in the eye and is known as the “Great Masquerader.” This makes diagnosis challenging, and it is now a common practice to test for syphilis in any case of infectious uveitis. Most cases of ocular syphilis present as uveitis, and the visual acuity depends on structures involved. It is a treatable disease with a good visual outcome; therefore, this study intends to describe the clinical profile and visual outcome after completing treatment in patients diagnosed with uveitic syphilis in Selayang Hospital, Malaysia.

## Materials and methods

We reviewed all retrospective cases diagnosed with ocular syphilis in a tertiary referral center from January 2014 to December 2019. A total of 31 cases were reviewed, and the collected data included demographic features, history of high-risk behavior, ocular symptoms and signs, visual acuity at presentation and after completing treatment, treatment received, complications, and human immunodeficiency virus (HIV) status. Serology tests for confirmation of the diagnosis were also included, such as rapid plasma reagin (RPR), venereal disease research laboratory (VDRL) titer, and treponema pallidum hemagglutination (TPHA) tests, and in some cases included the VDRL cerebrospinal fluid (CSF) test.

## Results

A total of 31 patients with ocular syphilis were identified within the study period, and 17 were found to have HIV co-infection with a CD4 cell count ranging from 91 to 328 cells/mm3. Male patients comprised the majority at 27 cases, while the remaining four patients were female. Of the 31 cases, 19 were under 50 years old, while the remaining 12 were 51 years old and over. Twenty one of the patients were ethnic Malay, six were ethnic Chinese, and four were ethnic Indian (Table 1). Twenty of the patients reported high-risk behaviors, and among them, six cases were HIV-infected homosexuals. The rest denied any high-risk behaviors.

**Table 1:** Demographic features and HIV status of 31 patients with ocular syphilis

	number of patients	percentage (%)
Gender		
Male	27	87%
Female	4	13%
Ethnicity		
Malay	21	68%
Chinese	6	19%
Indian	4	13%
HIV status		
HIV positive	17	55%
HIV negative	14	45%

**Figure 1:**
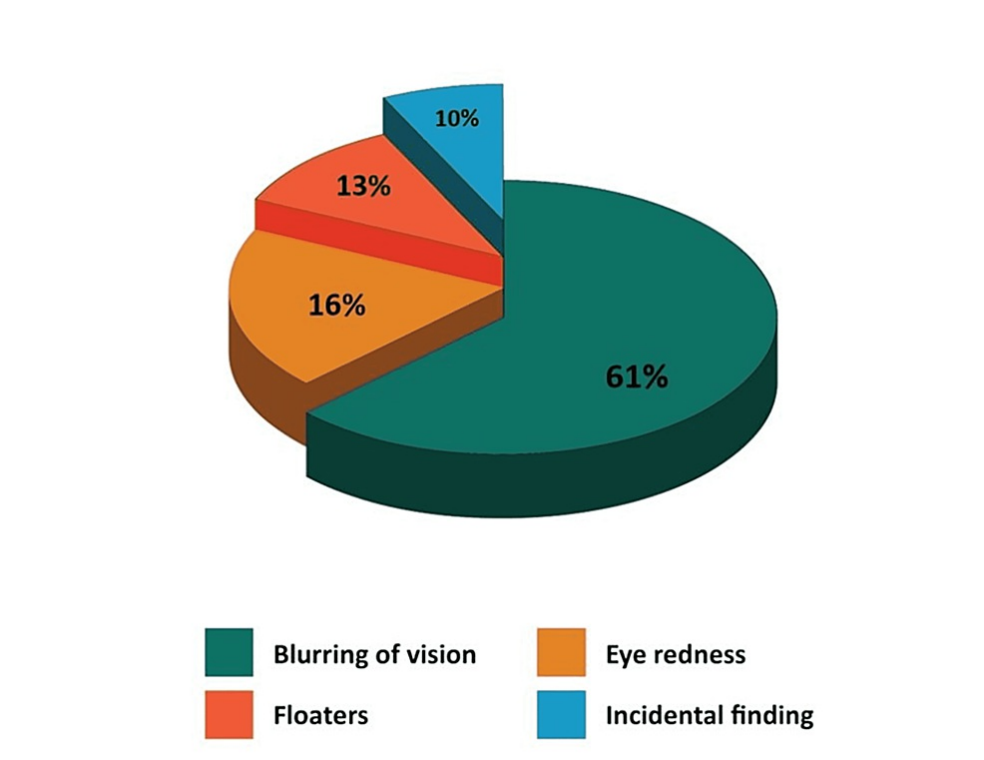
Presenting symptoms of ocular syphilis in this study

**Figure 2:**
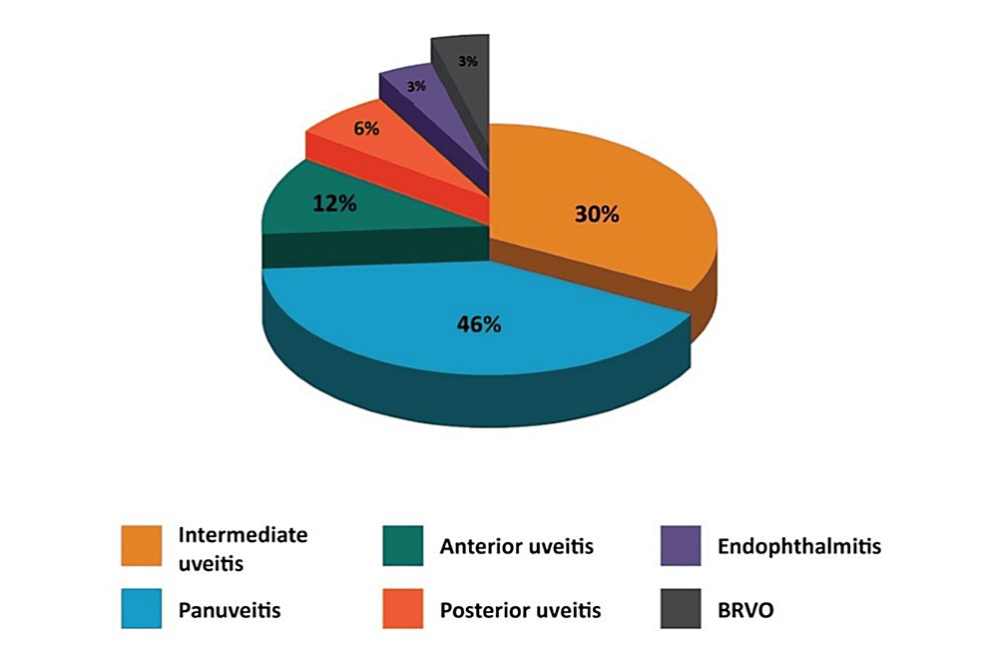
Clinical manifestations of ocular syphilis in this study BRVO: Branch retinal vein occlusion

The most common presenting symptoms were blurred vision (61%), eye redness (16%), floaters (13%), and incidental findings (10%) (Figure 1). The duration of symptoms ranged from one day to four months. There were 18 bilateral cases and 13 unilateral cases. The largest share of the cases presented as panuveitis (14 cases), followed by intermediate uveitis (nine cases), anterior uveitis (four cases), posterior uveitis (two cases), endophthalmitis (one case), and branch retinal vein occlusion (one case) (Figure 2). At least 22% of the cases presented with other secondary syphilis features such as diffuse maculopapular rash, including palms and soles, malaise, fever, and joint pain.

All patients had serology testing to confirm the diagnosis of syphilis. The non-treponemal test RPR and the treponemal test TPHA were carried out. Eleven of the patients were found to have an RPR titer of less than 1:4, and the remaining with RPR titer ranging from 1:8 to 1:1024. Only 12 patients consented to a lumbar puncture for the CSF VDRL test, and only one tested positive.

All patients received intravenous penicillin for 14 days, and none reported a Jarisch-Herxheimer reaction. Treatment was started based on the neurosyphilis protocol, which includes IV administration of 3.0 to 4.0 million units of benzylpenicillin every four hours for 14 days. In addition to receiving antibiotic therapy, three non-HIV-infected patients also received oral steroids as adjunctive therapy to control inflammation. 

All cases reported a good outcome with improvement in visual acuity of at least two Snellen lines after treatment. 

## Discussion

Syphilis is a sexually transmitted infection caused by spirochaete *treponema pallidum*. It is a disease that is transmitted via oral, anal and vaginal intercourse. It can also be transmitted from mother to fetus transplacentally. Cases in Malaysia have fluctuated from year to year, and limited data were available due to under-reporting. It is commonly associated with HIV infection, which therefore needs to be addressed as it may impose a burden on our healthcare system.

Ocular syphilis is a manifestation of chronic syphilis infection. It may occur at any stage and even be detected during the latent stage. Patients are usually asymptomatic, allowing the disease to progress without treatment. In our study, males are affected more than females. A similar study done in the United States by Oliver et al. reported a 14-fold greater number of ocular syphilis infection in male cases (362) compared to female cases (26), regardless of HIV status [1]. Another study by Moradi et al. at Johns Hopkins Hospital reported seven times more male patients, and Yap et al. in Singapore found 10 times more male patients than female [2,3]. They postulated that increased ocular syphilis cases in men were due to the practice of homosexuality.

In our study, 20 male patients claimed to have a history of unsafe sexual behavior, of which 11 were tested to be HIV-positive but denied any male-to-male sexual practices. Six patients identified themselves as homosexuals. These findings are expected, as homosexuality is considered a stigma in Malaysian culture. However, many studies done globally have shown that the incidence of HIV is growing more rapidly among MSM than in other subgroups. A study was done in Malaysia by Koh et al. observed a higher HIV prevalence amongst MSM compared to the non-MSM population [4]. Similar studies by Marcus et al. and Solomonet al., done in Europe and India, respectively, reported the same results [5,6].

Ocular syphilis is a great mimicker in patients presenting with uveitis. Our study found that the two most common presentations are panuveitis (46%) and intermediate uveitis (30%). This is also seen in studies by Yang et al. and Puech et al. [7,8]. The majority of our patients are HIV-positive (55%), and more than half (59%) presented with ocular syphilis before the diagnosis of HIV being made. This study also observed a higher risk of syphilis co-infection in HIV patients with CD4 cell counts <350 cells/ mm3, consistent with other studies [9,10]. This further emphasizes the presence of co-infection with other sexually transmitted diseases and the need to test for HIV in this group of patients. 

Overall, visual prognosis is good for patients with no underlying ocular comorbidity, and the results showed a positive visual outcome for those receiving treatment. It is a potentially sight-threatening disease, and with prompt treatment, the disease can be curable.

## Conclusions

Awareness of the serious ocular complications that may arise from symptoms that initially present as mild is important in those managing syphilis. Early detection and treatment will usually preserve visual acuity and ocular function. This study is also intended to create awareness among general practitioners to make an appropriate referral to an ophthalmologist when ocular syphilis is suspected. Additionally, it highlights the need for a high degree of suspicion of HIV co-infection, as the majority of our patients were discovered to be HIV-seropositive. Thus, HIV screening is mandatory in all patients presenting with syphilitic uveitis.
